# Teenage suicide cluster formation and contagion: implications for primary care

**DOI:** 10.1186/1471-2296-7-32

**Published:** 2006-05-17

**Authors:** Lars Johansson, Per Lindqvist, Anders Eriksson

**Affiliations:** 1 Section of Forensic Medicine, Dept. of Community Medicine and Rehabilitation, Umeå University, Sweden; 2 Division of Forensic Psychiatry, Dept. of Clinical Neuroscience, Karolinska Institute, Stockholm university, Sweden

## Abstract

**Background:**

We have previously studied unintentional as well as intentional injury deaths among teenagers living in the four northernmost counties, forming approximately 55% of Sweden with 908,000 inhabitants in 1991. During this work, we found what we suspected to be a suicide cluster among teenagers and we also suspected contagion since there were links between these cases. In this present study, we investigate the occurrence of suicide clustering among teenagers, analyze cluster definitions, and suggest preventive measures.

**Methods:**

A retrospective study of teenager suicides autopsied at the Department of Forensic Medicine in Umeå, Sweden, during 1981 through 2000. Police reports, autopsy protocols, and medical records were studied in all cases, and the police officers that conducted the investigation at the scene were interviewed in all cluster cases. Parents of the suicide victims of the first cluster were also interviewed. Two aggregations of teenager suicides were detected and evaluated as possible suicide clusters using the US Centers for Disease Control definition of a suicide cluster.

**Results:**

Two clusters including six teenagers were confirmed, and contagion was established within each cluster.

**Conclusion:**

The general practitioner is identified as a key person in the aftermath of a teenage suicide since the general practitioner often meet the family, friends of the deceased, and other acquaintances early in the process after a suicide. This makes the general practitioner suitable to initiate contacts with others involved in the well-being of the young, in order to prevent suicide cluster formation and para-suicidal activities.

## Background

Although our knowledge of teenager suicide behaviour is increasing, we lack sufficient knowledge of suicide clustering, which accounts for 1–13% of teen suicides and is two to four times more common among teenagers than in other age groups [1-3]. One possible mechanism behind suicide clustering is contagion, which may be transmitted by personal communication, through templates provided by teen icons and by the media. The contagious role of media in the alleged endemic spread of destructive behaviour has been debated in connection with school shootings in the US [4,5]. In addition, the influence of the Internet, its spread of information and its effect on suicidal teens, should be considered [6].

In order to disclose and describe possible clustering, this study investigates a cohort of teen suicides. Additionally, the role of contagion and of suicide cluster definitions is discussed in order to provide a basis for further research and preventive measures. Special emphasis is given to the preventive role of primary care concerning suicide and cluster formation among the young [cf [7]].

## Methods

All suicides (E950–E959; WHO 1977) from January 1, 1981 through December 31, 2000 (n = 88) among individuals aged 13 through 19 years at death were identified using the databases of the Department of Forensic Medicine in Umeå, National Board of Forensic Medicine. The data covers the four northernmost counties of Sweden (908,000 inhabitants, 1991). The reliability of these databases was checked by comparing it to the National Cause of Death Register. No additional cases were added [cf [8]].

The original material comprised of police reports, autopsy protocols, and medical records if available. Two of the authors also interviewed the investigating police officers of the suicides occurring from January 1993 through May 1995 [8]. According to Swedish laws and regulations, the investigation of all unnatural deaths should include a medico-legal autopsy [cf [9]]. For the cases from 1995 through 1996, the parents as well as the police officers were interviewed.

Toxicological data refer to the results from analyses of femoral vein blood. The analyses were conducted at the Department of Forensic Chemistry, Linköping, Sweden.

We used the Centers for Disease Control definition of a suicide cluster: "a group of suicides or suicide attempts, or both, that occurs closer together in time and space than would normally be expected in a given community", but excluded "suicide attempts" since we did only have anecdotal information on such [10].

The regional ethical review board in Umeå approved of the procedures used, given that no single case could be identified at publication.

## Results

### The original material

Eighty-eight teenagers (66 boys, 22 girls) committed suicide with an annual rate of 0–8 cases. The lowest numbers of suicides were found among the 13 year-olds (1 case), and the highest among the 18 year-olds (26 cases).

The suicide methods most commonly used were hanging (n = 35), shooting (n = 26), intoxication (n = 14), and jumping from a high place (n = 5). Fifty-six (64%) tested negative for blood alcohol, 30 (34%) tested positive with a blood alcohol concentration from 0.2 g/l to 2.9 g/l, and two were not tested.

Two possible clusters, each including three victims, were identified. All cluster cases were ethnic Swedes and were raised by one or both of their biological parents.

### First cluster

Three teenagers who knew each other (according to both their parents and the investigating police officers) committed suicide by hanging within an 11-month period. Two lived in the same industrial community adjacent to a city (with a population of approximately 40,000) where the third victim lived.

The first case was a 17-year-old boy who regularly attended a church to which the parents of the third suicide victim belonged. His parents perceived him as being "depressed" the last few months before the suicide, and they noticed that he was becoming increasingly "unrealistic" in his thinking. He consulted a school nurse due to a headache a few weeks before the suicide, but he never received psychiatric treatment. Post-mortem analyses were negative for alcohol and illicit drugs.

A 17-year-old girl committed suicide eight months later. She lived and worked close to where the first case lived. She did not exhibit problems at school and had many friends. After her death, the parents recalled that she appeared calmer the last month of her life. She took better care of her personal belongings, spent more time with her family, and turned to her childhood friends again. Post-mortem analyses were negative for alcohol and illicit drugs.

The third teenager, a 14-year-old girl, committed suicide eleven months after the first victim. Her parents were members of the same church as the first case. The mother of the first case described this girl's parents as the spiritual parents of her son. The girl knew him as a friend and she expressed suicidal thoughts almost from the day he committed suicide. About one month after his suicide, the girl's parents noticed that she was talking to herself, hallucinating, and verbalising obsessive thoughts. For several months, she was openly threatening to commit suicide and her father hid ropes and other possible suicide means. The parents contacted a child and adolescent psychiatric clinic, and they attended several out patient sessions with a psychologist. There was a history of psychotic disease in the family. Post-mortem analyses were negative for alcohol and illicit drugs.

### Second cluster

Three teenagers committed suicide by jumping from a tower and by hanging within a 17-month period. They lived on the same block in the same city (with a population of approximately 100,000), and they knew each other according to the investigating police officers. No one was known to abuse alcohol or other drugs.

The first case was an 18-year-old boy who had long-lasting problems with his schoolmates. He spent more time at home before the suicide and was described by his parents as reserved. Some time before the suicide he disappeared from home and his concerned parents found him near the tower from which he later leaped. The post-mortem blood alcohol concentration was 1.5 g/l.

A 17-year-old boy was the second victim 14 months later. He jumped from the same high place as the previous case. He was uneasy with school, and his parents had noticed that he had been quieter the last few days before the suicide. He told them that he was going to visit a friend, but instead he climbed a fence to get access to the tower and he left a suicide note on the tower. His post-mortem blood tested positive for tetrahydrocannabinol.

The third case, a 16-year-old girl, committed suicide three months later. She identified the second victim by name in her suicide note, saying she was now going to talk to him. During her last year, she had an intense interest in suicide-related information such as newspaper articles about suicide and music by artists who had committed suicide. Her family had noticed that she had become more introverted the last weeks before her suicide. Her father received a telephone call from a school counsellor on the day of the suicide to inform him that his daughter was suicidal and that they should call the child and adolescent psychiatric clinic first thing in the morning. The father rushed home; he arrived too late. Post-mortem analyses were negative for alcohol and illicit drugs.

## Discussion

We identified two suicide clusters in two different communities. The suicides occurred within a geographical and timely proximity in each cluster. The teenagers knew one another and there were striking similarities between the cases. This made us believe that suicide contagion contributes to some teenager suicides.

The psychological and social impact of a teenager suicide on the family and society is immeasurable. On average, one single suicide intimately affects at least six other people and if it occurs in a school, it impacts hundreds of people [11]. General practitioners, social workers, and school staff should be involved in the tragic aftermath of every teenager suicide. The importance of a raised awareness of the risk of suicide clustering after any teenager suicide can not be stressed enough. This awareness may prevent further suicides when primary prevention has failed.

Contagion is one mechanism behind cluster formation; however, the significance of such contagion is controversial. A Finnish study supports the contagion hypothesis, whereas another questions it [12,13]. Another possible mechanism is that exposure to suicide may induce or exacerbate depression in vulnerable adolescents, a vulnerability that may manifest as a suicidal behaviour through the mechanism of complicated bereavement rather than through contagion and imitation [14]. Either way, it is important to bear in mind the risk of further suicides and the risk of cluster formation in a society struck by a teenager suicide [2,3].

### Definitions

Different definitions of suicide clusters with respect to the required number of suicides and geographic and temporal limitations have been used [10,15-17]. Another view is that the most relevant issue is whether "a community perceives that it is experiencing a suicide cluster" or not because the perception of suicide clustering in itself may increase the risk of imitation and contagion [18]. Obviously, this lack of a standardised operational definition limits comparability of different investigations [15,19].

In addition to the various definitions of a cluster, there are also different types of clusters. We prefer to distinguish between *statistical *clusters and *contagious *clusters. In a *statistical cluster*, a predefined number of cases cannot be a part of the definition because clustering will depend on the suicide base rate and the size of the population under study. To define a cluster, the rise must be statistically significant, not merely any rise above the mean. Different statistical methods have been used [2,15,19,20]. The disadvantage of using statistical surveillance is that it can detect increasing suicide rates and cluster formation only retrospectively, and cannot be used for the purpose of intervention [18].

In a *contagious cluster*, we suggest that the number of cases should be three or more. Delimitation of a geographic area is of less importance due to today's information technology. For example, a news story of a celebrity's death can spread all over the world within minutes, initiating contagion and increasing self-destructive behaviour and suicidal activities among those who identify themselves with the celebrity [21,22].

Finally, we suggest that only deaths as the result of suicide should be part of a definition of a *suicide cluster*. The term *cluster of self-destructive behaviour *is a more suitable term when also suicide attempts are included.

### Postvention and prevention

Suicide cluster formation can be described metaphorically as a contagious "disease". When susceptible individuals with this "disease" come together, they might super-infect one another, resulting in a suicide cluster. Community surveys have demonstrated that approximately one quarter of adolescents have experienced suicidal thoughts, which serves as a breeding ground for contagion [23,24].

The number of contagious cases may be reduced by the following measures: identifying the individuals most susceptible to contagion, such as close friends; identifying individuals who have attempted suicide and/or those known to have psychiatric problems, especially boys; and by defusing the tension built up in the community after a suicide [10,12,14,16,23,25-28]. According to Shneidman [29], the most important role of *postvention *(i.e., actions taken *after *a suicide) is to decrease the emotional distress of affected individuals. Recommendations for a community plan to contain suicide clusters have been issued, but the risk of increased suicidal ideation after improper postvention must be acknowledged [10,25,27].

The general practitioner is often the physician who has to confirm the death of a suicide victim and who at an early stage will meet the family, friends, and others involved in the aftermath of a teenager suicide. This position makes the general practitioner suitable to make contacts for initiation of postventive actions in the stricken community and the general practitioner should also be used as a resource concerning primary prevention of suicide. The World Health Organization has identified general practitioners as a particularly relevant group to the prevention of suicide [11]. A Danish study on contacts with the health care system prior to suicide showed that almost 70% of those who had committed suicide had been in contact with their general practitioner during the last month before their suicide [30]. This shows the potential of the general practitioner as a key person in suicide prevention, but also indicates the difficulties in identifying individuals at risk. In some parts of Sweden, e.g., the southern parts of Lapland, psychiatrists from the local hospital visits primary care centres on regular basis. Close cooperation between general practitioners and psychiatrists could facilitate the clinical assessment and also enhance the possibilities of identifying those at risk.

### Validity

A partially retrospective study like this, relying mainly on file data, is liable to miss relevant data such as intimate personal bonds between subjects. To some degree, we have compensated this by interviewing the investigating police officer and, in the first cluster, also the parents of the suicide victim. In total, this study represents minimum data, data that only conveys the tip of the iceberg, and it is also important to remember that cluster formation can occur as para-suicidal and self-destructive clusters. We obtained anecdotal information on both para-suicidal and self-destructive behaviour during the interviews, but did not investigate it further within the frames of this study.

## Conclusion

There are still many questions and few answers in the field of suicide prevention. School staff, police officers, social workers, primary care officials, and others involved in the well-being of the young should be aware of the risk of cluster formation, which could prevent further suicides.

## Competing interests

The author(s) declare that they have no competing interests.

## Authors' contributions

Lars Johansson participated in the design of the study and interviewed the investigating police officers and prepared the manuscript.

Per Lindqvist participated in the design of the study and interviewed the parents of cluster one and have read and commented on the manuscript.

Anders Eriksson participated in the design of the study and have read and commented on the manuscript.

All authors read and approved the final manuscript.

## Pre-publication history

The pre-publication history for this paper can be accessed here:



## References

[B1] Pelkonen M, Marttunen M (2003). Child and adolescent suicide. Epidemiology, risk factors and approaches to prevention. Paediatr Drugs.

[B2] Gould MS, Wallenstein S, Kleinman MH (1990). Time-space clustering of teenage suicide. Am J Epidemiol.

[B3] Gould MS, Wallenstein S, Kleinman MH, O'Carroll P, Mercy J (1990). Suicide clusters: An examination of age-specific effects. Am J Public Health.

[B4] Cohen A Criminals as copycats. Time Magazine.

[B5] Texeira E, Krikorian G, Martelle S 5 hurt in gunfire at high school near San Diego; student is held. Los Angeles Times.

[B6] Gallagher KE, Smith DM, Mellen PF (2003). Suicidal asphyxiation by using pure helium gas. Case report, review and discussion of the influence of the Internet. Am J Forensic Med Pathol.

[B7] Stanistreet D, Gabbay MB, Jeffrey V, Taylor S (2004). The role of primary care in the prevention of suicide and accidental deaths among young men: an epidemiological study. Br J Gen Pract.

[B8] Lindqvist P, Johansson L (2000). Teenage suicides in northern Sweden: An interview study of investigating police officers. Inj Prev.

[B9] Öström M, Ahlm K, Eriksson A (2001). Problematic management of unnatural deaths. Läkartidningen.

[B10] O'Carroll PW, Mercy JA, Steward JA (1988). CDC recommendations for a community plan for the prevention and containment of suicide clusters. Morb Mortal Wkly Rep.

[B11] World Health Organization (2000). Preventing suicide. A resource for general physicians. Geneva.

[B12] Poijula S, Wahlberg KE, Dyregrov A (2001). Adolescent suicide and suicide contagion in three secondary schools. Int J Emerg Ment Health.

[B13] Joiner TE (1999). The clustering and contagion of suicide. Current Directions in Psychological Science.

[B14] Brent DA, Perper JA, Moritz G, Allman C, Schweers J, Roth C, Balach L, Canobbio R, Liotus L (1993). Psychiatric sequelae to the loss of an adolescent peer to suicide. J Am Acad Child Adolesc Psychiatry.

[B15] Gould MS, Wallenstein S, Davidson L (1989). Suicide clusters: A critical review. Suicide Life Threat Behav.

[B16] Davidson LE, Rosenberg ML, Mercy JA, Franklin J, Simmons JT (1989). An epidemiologic study of risk factors in two teenage suicide clusters. JAMA.

[B17] Hazell P (1993). Adolescent suicide clusters: Evidence, mechanisms and prevention. Aust N Z J Psychiatry.

[B18] O'Carroll PW, Mercy JA (1990). Responding to community-identified suicide clusters: statistical verification of the cluster is not the primary issue. Am J Epidemiol.

[B19] Gibbons RD, Clark DC, Fawcett J (1990). A statistical method for evaluating suicide clusters and implementing cluster surveillance. Am J Epidemiol.

[B20] Wallenstein S, Gould MS, Kleinman M (1989). Use of the scan statistic to detect time-space clustering. Am J Epidemiol.

[B21] Hawton K, Harriss L, Simkin S, Juszczak E, Appelby L, McDonnell R, Amos T, Kiernan K, Parrott H (2000). Effect of death of Diana, Princess of Wales on suicide and deliberate self-harm. Br J Psychiatry.

[B22] Jobes DA, Berman AL, O'Carroll PW, Eastgard S, Knickmeyer S (1996). The Kurt Cobain suicide crisis: Perspectives from research, public health, and the news media. Suicide Life Threat Behav.

[B23] van Heeringen C (2001). Suicide in adolescents. Int Clin Psychopharmacol.

[B24] Miotto P, De Coppi M, Frezza M, Petretto D, Masala C, Preti A (2003). Suicidal ideation and aggressiveness in school-aged youths. Psychiatry Res.

[B25] Adler RS, Jellinek MS (1990). After teen suicide: Issues for pediatricians who are asked to consult to schools. Pediatrics.

[B26] Brent DA, Kerr MM, Goldstein C, Bozigar J, Wartella M, Allan M (1989). An outbreak of suicide and suicidal behavior in a high school. J Am Acad Child Adolesc Psychiatry.

[B27] Callahan J (1996). Negative effects of a school suicide postvention program – A case example. Crisis.

[B28] Shaffer D, Garland A, Gould M, Fisher P, Trautman P (1988). Preventing teenage suicide: A critical review. J Am Acad Child Adolesc Psychiatry.

[B29] Shneidman ES (1981). Suicide thoughts and reflections, 1960–1980: Postvention: The care of the bereaved. Suicide Life Threat Behav.

[B30] Andersen UA, Andersen M, Rosholm JU, Gram LF (2000). Contacts to the health care system prior to suicide: a comprehensive analysis using registers for general and psychiatric hospital admissions, contacts to general practitioners and practising specialists and drug prescription. Acta Psychiatr Scand.

